# A Chemically Defined, Xeno- and Blood-Free Culture Medium Sustains Increased Production of Small Extracellular Vesicles From Mesenchymal Stem Cells

**DOI:** 10.3389/fbioe.2021.619930

**Published:** 2021-05-26

**Authors:** Aliosha I. Figueroa-Valdés, Catalina de la Fuente, Yessia Hidalgo, Ana María Vega-Letter, Rafael Tapia-Limonchi, Maroun Khoury, Francisca Alcayaga-Miranda

**Affiliations:** ^1^Cells for Cells, Santiago, Chile; ^2^Consorcio Regenero, Chilean Consortium for Regenerative Medicine, Santiago, Chile; ^3^Laboratory of Nano-Regenerative Medicine, Centro de Investigación e Innovación Biomédica (CiiB), Universidad de los Andes, Santiago, Chile; ^4^School of Medicine, Faculty of Medicine, Universidad de los Andes, Santiago, Chile

**Keywords:** small extracellular vesicle, exosome, culture medium, xeno-free cell culture, blood-free cell culture, serum-free cell culture, chemically defined, mesenchymal stem (stromal) cells

## Abstract

Cell therapy is witnessing a notable shift toward cell-free treatments based on paracrine factors, in particular, towards small extracellular vesicles (sEV), that mimic the functional effect of the parental cells. While numerous sEV-based applications are currently in advanced preclinical stages, their promised translation depends on overcoming the manufacturing hurdles posed by the large-scale production of purified sEV. Unquestionably, the culture medium used with the parental cells plays a key role in the sEV’s secretion rate and content. An essential requisite is the use of a serum-, xeno-, and blood-free medium to meet the regulatory entity requirements of clinical-grade sEV’s production. Here, we evaluated Oxium^TM^EXO, a regulatory complying medium, with respect to production capacity and conservation of the EV’s characteristics and functionality and the parental cell’s phenotype and viability. A comparative study was established with standard DMEM and a commercially available culture medium developed specifically for sEV production. Under similar conditions, Oxium^TM^EXO displayed a three-fold increase of sEV secretion, with an enrichment of particles ranging between 51 and 200 nm. These results were obtained through direct quantification from the conditioned medium to avoid the isolation method’s interference and variability and were compared to the two culture media under evaluation. The higher yield obtained was consistent with several harvest time points (2, 4, and 6 days) and different cell sources, incluiding umbilical cord-, menstrual blood-derived mesenchymal stromal cells and fibroblasts. Additionally, the stem cell phenotype and viability of the parental cell remained unchanged. Furthermore, Oxium^TM^EXO-sEV showed a similar expression pattern of the vesicular markers CD63, CD9, and CD81, with respect to sEV derived from the other conditions. The *in vitro* internalization assays in different target cell types and the pharmacokinetic profile of intraperitoneally administered sEV *in vivo* indicated that the higher EV production rate did not affect the uptake kinetics or the systemic biodistribution in healthy mice. In conclusion, the Oxium^TM^EXO medium sustains an efficient and robust production of large quantities of sEV, conserving the classic functional properties of internalization into acceptor target cells and biodistribution *in vivo*, supplying the amount and quality of EVs for the development of cell-free therapies.

## Introduction

The therapeutic effects of mesenchymal stem/stromal cells (MSCs) are predominantly based on their secretome, consisting of bioactive secretion of factors and, notably, extracellular vesicles (EV) (Camussi et al., 2013; Pegtel and Gould, 2019; Witwer et al., 2019). Currently, about 1,175 MSC-related clinical trials are listed in the NIH clinical trial database (search carried out in September 2020)^1^. A few dozen cell-based therapies have obtained market authorization in several countries (Cuende et al., 2018), and at least another dozen of approved MSC-based therapies are expected to reach the market by the year 2030 (Olsen et al., 2018). We have recently demonstrated (Kurte et al., 2020) that MSC’s high immunoplasticity depends on the exposure duration with the inflammatory milieu, leading into either an enhanced or an impairment therapeutic activity, a matter of great concern for their clinical use. Both the translational advances and the limitation of the use of MSC in some clinical applications have pushed the field toward exploring their therapeutic potential without the need for cell transplantation.

Small extracellular vesicles (sEV) are non-self-replicative lipid-based vesicles secreted by virtually all types of cells under both physiological and pathological conditions (Kalluri and LeBleu, 2020). Unlike cell-based therapy, the use of sEV therapeutics is free of safety concerns related to uncontrolled cell division and immune rejection (Alcayaga-Miranda et al., 2016; Baharlooi et al., 2020). sEV are characterized by bodies smaller than 200 nm in diameter with tetraspanins CD63, CD81, and CD9 present in their membrane (Théry et al., 2018; Witwer et al., 2019). The innovative cell-free strategy based on the application of EV provides the functional effect of the parental stem cell without the negative influence of the pathological environment on their secretion profile. Their role in regenerative medicine is based on the fact that they could “mediate most of beneficial regenerative effects of MSCs without possible side effects of using MSCs themselves” (Sagaradze et al., 2018), showing similar or even superior therapeutic capacity than the treatment with their parental MSCs (Willis et al., 2017). Considering the burst of interest in their biological effects, the use of sEV as cell-free therapy is widely under investigation with promissory preclinical results (Lener et al., 2015; Alcayaga-Miranda et al., 2016; Rosenberger et al., 2019). Moreover, the choice of using sEV over liposomes and other artificial nanoparticles such as nanocarriers has been backed by their higher stability (Askenase, 2020). Currently, only a handful of sEV-based therapeutics have evolved to a state that is mature enough for clinical evaluation (Zipkin, 2019). When products retain uncertainties regarding early steps of development and manufacturing validation, such as reagent use and procedures, their clinical application is faced by regulatory hurdles and reluctant sponsors, hindering their translational pathway (Mastrolia et al., 2019).

The cell culture industry, considered as the main pillar of the biopharmaceutical market, is witnessing a drift away from fetal bovine serum (FBS)-based formulations, favoring the development of chemically defined media, especially for clinical-grade cultures (Kalorama Information, 2018). According to regulatory agencies, manufacturers of human biological medicinal products must favor the use of non-ruminant material in order to avoid the risky use of potentially infectious materials (United States Food and Drug Administration, 2020). Their guidelines propose the use of human platelet lysate (hPL) as an alternative to FBS (European Medicines Agency, 2013; Guiotto et al., 2020). Therefore, new media formulations containing xeno- and blood-free components are required to circumvent the regulatory restrictions, ensuring at the same time the performance consistency in cell culture media. Amidst the cells with therapeutic potential, MSCs are considered as “a critical raw material for regenerative medicine products, including cell-based therapies, engineered tissues, or combinations products” (Olsen et al., 2018).

Since FBS or hPL are supplements rich in their own sEV, the production of both research and clinical-grade sEV must be carried out in exogenous sEV-free medium in order to avoid the contamination of the produced and the exogenous sEV. The use of a serum-depleted medium or medium without FBS (often called “serum starvation”) provides an undesired stress environment, which is suboptimal for cell growth and viability, and therefore an undesirable reduction in the secretory rate of sEV to the supernatant (Lehrich et al., 2018; Haraszti et al., 2019). Furthermore, the generated oxidative-stress products can be shuttled within the sEV cargo, leading to important concerns for functional changes and adverse effects. In order to reduce time and resources needed to produce a therapeutic dose, new compliant media are required as the central part of the GMP-compliant manufacturing strategy for increased and reproducible sEV production. A defined and consistent protocol devoid from contaminant and oxidative stress agents will fulfill the quality-control requirements necessary for batch releases, regulation fulfillment, and the taking of sEV’s advantages, such as low toxicity, biocompatibility, biological permeability/distribution, ease of handling and storage, and the possibility of loading them in order to use them as drug-delivery vehicles (Roura and Bayes-Genis, 2019; Zipkin, 2019).

Here, we report the use of a new serum-, xeno-, and blood-free medium (Oxium^TM^EXO), tested for the production of extracellular vesicles from umbilical cord-derived human MSCs (UC-MSCs), menstrual blood-derived human MSCs (Mens-MSCs), and fibroblasts. UC-MSCs were chosen because previous work in our laboratory showed that these cells exhibit higher clonogenic, proliferative, and migration potential than bone marrow-derived MSCs (BM-MSCs) and enhanced the secretion of chondrogenic factors (González et al., 2015; Bartolucci et al., 2017). The latter led to probe and demonstrate their safety and efficacy as cell therapy for knee osteoarthritis treatment (Park et al., 2017; Matas et al., 2019). In a similar way, our laboratory and others had demonstrated several therapeutic effects of Mens-MSCs, including antitumor properties (Alcayaga-Miranda et al., 2016; Chen et al., 2019; Rosenberger et al., 2019), and their superiority with respect to several functional aspects in comparison with BM-MSCs (Alcayaga-Miranda et al., 2015a), making them interesting candidates for research in cell-based or cell-free cancer treatments, research that is still ongoing today in our lab. Finally, fibroblasts were used as a non-MSC cell lineage control. Compared to standard (DMEM) and commercially available medium, the cell culture in Oxium^TM^EXO showed a superior performance in terms of sEV-production numbers while maintaining MSCs and sEV characteristics *in vitro* and *in vivo*. Oxium^TM^EXO can represent an alternative to produce sEV from tissue-derived human MSCs, applicable from the bench to a large-scale platform, while maintaining cell phenotype and multipotency potential of the sEV cell source.

## Materials and Methods

### Ethics Statement

Menstrual blood and umbilical cord were collected from healthy donors, and osteoarthritis (OA) cartilage was obtained from patients undergoing hip surgery. All tissue samples were collected after written informed consent following institutional guidelines and ethical committee approval. All animal studies were performed at the Cells for Cells Animal Facility in accordance with protocols revised and approved by the Institutional Animal Care and Use Committee of Universidad de los Andes.

### Cell Culture, MSC Characterization, and hPL Preparation

Mesenchymal stromal cells (MSCs) were isolated, characterized, cultured, and expanded as we previously described (Alcayaga-Miranda et al., 2015a,b; Bartolucci et al., 2017; Matas et al., 2019) and cryopreserved at low passage (<5) until use. Briefly, cells were cultured in a maintenance medium composed of Dulbecco’s modified Eagle’s medium (DMEM), high glucose, supplemented with 1% penicillin/streptomycin solution (10,000 U/mL and 10,000 μg/mL, respectively), 1% L-glutamine (200 mM) (all from Gibco, Paisley, United Kingdom), and 5% human platelet lysate (hPL).

All MSCs were characterized according to the guidelines of the International Society for Cell and Gene Therapy (ISCT) (Dominici et al., 2006). The trilineage differentiation capacity of UC-MSCs cultured for 6 days in DMEM, Oxium^TM^EXO, or commercial medium was evaluated using the StemPro^TM^ differentiation kits (Gibco, Life Technologies, New York, NY, United States) in accordance with the manufacturer’s instructions with some modifications. In brief, to induce osteogenic differentiation, cells were grown at 5 × 10^4^ cells/cm^2^ with StemPro^TM^ Osteogenesis Differentiation Kit (Cat. #A1007201). After 14 days, calcium deposits were detected by Alizarin Red staining (Sigma-Aldrich, Merck, St. Louis, MO, United States, Cat. #A3757). To induce adipogenic differentiation, cells were incubated with StemPro^TM^ Adipogenesis differentiation kit (Cat. #A1007001) medium at 1 × 10^4^ cells/cm^2^. After 14 days, cell differentiation into adipocytes was confirmed by Oil Red O staining of lipidic vacuoles (Sigma-Aldrich, Merck, St. Louis, MO, United States, Cat. #O0625). For chondrogenic differentiation, cells were incubated at 1.7 × 10^5^ cells/μL in 10 μL of culture medium for 1 h to favor micromass formation. Then, cells were cultured in StemPro^TM^ Chondrogenesis differentiation kit (Cat. #A1007101) differentiation medium according to the manufacturer’s instructions for 21 days, assessing chondrogenic differentiation with Safranin O staining (Sigma-Aldrich, Merck, St. Louis, MO, United States, Cat. #S2255). Immunophenotyping of MSCs was performed by staining with monoclonal antibodies against CD105 (Cat. #560819), CD90 (Cat. #555596), CD73 (Cat. #561258), HLA-DR-DP-DQ (Cat. #555558), CD34 (Cat. #555824), CD19 (Cat. #644491), CD14 (Cat. #555398), and CD45 (Cat. #5554829) (all from BD Pharmingen, San Diego, CA, United States) using standard protocol. The staining was performed for 20 min at 4°C in darkness, and the dead cells were discarded using Live/Dead fixable yellow stain (Life Technologies, Carlsbad, CA, United States, Cat. #L34968). The analysis was performed by flow cytometry using a FACSCanto^TM^ II cytometer (BD Biosciences, San Jose, CA, United States). The data acquired were analyzed using the FlowJo software V10 (Tree Star, Ashland, OR, United States). The analysis was performed on a minimum of three different cell cultures with cells at passage 5.

To prepare hPL, human-donor platelets (*n* = 20) were obtained from a blood bank using the platelet apheresis method. hPL was prepared in accordance with a previously described method with some modifications (Burnouf et al., 2016). Briefly, 20-donor pooled groups of platelets were thawed at 37°C for 3 h and then frozen at −80°C overnight. The thaw-and-freeze steps were repeated two times. To remove membrane fragments, the lysate was centrifuged at 13,000 *g* at 4°C for 20 min and the supernatant was filtered through a 40-μm cell strainer (Falcon, Corning, Tewksbury, MA, United States, Cat. #352340). For the depletion of fibrinogen, 10% w/v sterile CaCl_2_ (Laboratorio Sanderson, Santiago, Chile, Sanitary Registration #F13540/14) was added to a final concentration of 10 mM. The solution was incubated at 37°C for 2 h to allow the formation of fibrinogen clot, then vortexed for the disruption of the clot and centrifuged at 13,000 *g* for 15 min at 4°C. The supernatant was filtered in a 40-μm cell strainer, mixed, and aliquoted to freeze at −80°C until use.

Chondrocytes were isolated, characterized, cultured, and expanded as previously described (Rackwitz et al., 2014). Human MSCs and human chondrocytes were donated by Cells for Cells (Las Condes, Santiago, Chile)^2^. Normal human dermal fibroblasts were purchased from Lonza (Walkersville, MD, United States, Cat. #CC-2511), and the metastatic human breast cancer MD-MB-231 cell line was obtained from the American Type Culture Collection (Manassas, VA, United States, Cat. #HTB-26^TM^) and cultured according to the manufacturer’s protocol. All cells were maintained in a humidified incubator (37°C; 5% CO_2_) and regularly tested for mycoplasma contamination using a PCR detection kit (Applied Biological Materials Inc., Richmond, BC, Canada, Cat. #G238) according to manufacturer’s instructions.

### Apoptosis Assay

The cellular apoptosis was evaluated following the protocol as previously described by our group (Rosenberger et al., 2019). Briefly, 6,250 cells/cm^2^ were seeded in 100 mm plates (Falcon, Franklin Lakes, NJ, United States, Cat. #353003) in maintenance medium. After 48 h, the culture medium was removed, and the cells were washed three times with phosphate-buffered saline (PBS 1×) before starting the culture in the different induction medium: (a) DMEM high glucose + 1% L-glutamine; (b) Oxium^TM^EXO (Consorcio Regenero S.A., Las Condes, Santiago, Chile; patent No. PCT/CL2019/100175, Tapia-Limonchi et al., 2019); or (c) commercial medium (RoosterBio Inc., Frederick, MD, United States, Cat. #M2001). After 6 days, cell supernatants were mixed with the trypsinized cells in order to include detached dead cells in the analysis. Then, cells were stained with Annexin V-APC (BioLegend, San Diego, CA, United States, Cat. #640920) and 7-aminoactinomycin D (7-AAD) (BioLegend, San Diego, CA, United States, Cat. #420403) in Annexin V binding buffer (BioLegend, San Diego, CA, United States, Cat. #422201). The analysis was performed by flow cytometry using a FACSCanto^TM^ II cytometer (BD Biosciences, San Jose, CA, United States). The data acquired were analyzed using the FlowJo software V10 (Tree Star, Ashland, OR, United States).

### Small Extracellular Vesicle Production, Isolation, Characterization, and Staining

Small extracellular vesicles were produced and purified as previously described by our group with some modifications (Alcayaga-Miranda et al., 2016; Lopez-Verrilli et al., 2016; Rosenberger et al., 2019). Briefly, UC-MSC cells in passage 5 were seeded and expanded in a maintenance medium on three 10-layer Nunc^TM^ EasyFill^TM^ Cell Factory^TM^ systems (Thermo Fisher Scientific, Waltham, MA, United States, Cat. #140400) with a density of 6,250 cells/cm^2^. After cells reached ∼70% confluence, the maintenance medium was discarded and cells were washed three times with PBS 1× before addition of the induction media for sEV production: (a) DMEM high glucose + 1% L-Glutamine; (b) Oxium^TM^EXO (patent No. PCT/CL2019/100175); or (c) commercial medium (RoosterBio Inc., Frederick, MD, United States, Cat. #M2001). After 6 days, supernatants were collected and divided into two (in order to achieve two independent sEV’s isolations per medium), subjected to serial centrifugations of 600 and 2,000 *g* for 10 min at 4°C and sequential filtration with 0.45- and 0.22-μm pore-size PVDF membranes, to later be subjected to ultracentrifugation (Thermo Electron LED GmbH, Langenselbold, Germany, model Sorvall WX+) at 100,000 *g* for 70 min at 4°C in a swinging bucket rotor (Thermo Fisher Scientific, Waltham, MA, United States, Model TH-641). The pellet obtained was resuspended in approximately 100 μl of PBS 1× and stored at −80°C until use. A diagram of the protocol for cell cultures for sEV production and isolation is shown in Supplementary Figure 1.

Nanoparticle tracking analysis (NTA) was performed on a NanoSight NS300 system (Malvern Instruments Limited, Worcestershire, United Kingdom) to determine particle concentration and size distribution following the manufacturer’s instructions. Briefly, sEV fractions were processed in duplicate and diluted with PBS 1× over a range of concentration to obtain between 10 and 100 particles per image. sEV samples were mixed before the analysis. Five videos of 60 s each per sample were captured (camera level = 8), processed (detection threshold = 3), and analyzed to give the mean and mode of the particle’s size, together with a total particle concentration. Further analyses of the collected data allowed the determination of particle concentration according to different size ranges of interest: 0–50 nm; 51–200 nm; 201–300 nm, and those over 301 nm.

Small extracellular vesicles characterization was performed following the International Society for Extracellular Vesicles guidelines (Théry et al., 2018). The evaluation of surface markers of isolated sEV was done as described previously with some modifications (Suárez et al., 2017; Mendt et al., 2018). Briefly, 1.4 × 10^9^ particles resuspended in PBS 1× (400 μL) were incubated with Aldehyde/Sulfate Latex beads (1 μL) (Molecular Probes, Eugene, OR, United States, Cat. #A37304) in a rotatory mixer for 10 min at room temperature (RT). After the addition of PBS 1× (final volume of 800 μL), samples were incubated overnight in a rotatory mixer at 4°C. Four hundred microliters of 1 M glycine (0.33 M final concentration; United States Biological, Salem, MA, United States, Cat. #G8160) was added to the samples and incubated through continuous mixing for 1 h at RT. The samples were centrifuged at 8,000 *g* for 2 min at 4°C, and the pellet was resuspended in 100 μL 10% w/v Bovine Serum Albumin (BSA; Winkler Ltda., Santiago, Chile, Cat. #BM-0150) prepared in PBS 1× and incubated with continuous mixing for 45 min at RT. Then, the pellet was resuspended in 10 μL of 2% BSA solution containing separately the primary antibodies (0.5 μL) mouse α-human CD63 (Cat. #556019), CD81 (Cat. #555675), and CD9 (Cat. #555370) (all from BD Pharmingen San Diego, CA, United States) or containing the isotype control (5 μL) mouse IgG_1_ (BD Biosciences, San Jose, CA, United States, Cat. #349040); the incubations were performed under continuous mixing for 30 min at RT. The immunolabeled particle-coupled beads were washed once with PBS 1× and incubated with 25 μL of 10% BSA solution for 30 min at RT, to carry out a second wash step with PBS 1×. The pellet was resuspended in 10 μL solution containing 2% BSA and 0.5 μL of secondary antibody α-mouse IgG_1_ Alexa Fluor 488 (BioLegend, San Diego, CA, United States, Cat. #406626) and incubated for 30 min at RT. Finally, the sample was washed three times with PBS 1× and the pellet was resuspended in 100 μL of PBS 1× for the acquisition on the cytometer. The samples were analyzed on the cytometer FACSCanto^TM^ II cytometer (BD Biosciences, San Jose, CA, United States) and were recorded with at least 1 × 10^5^ events of beads. The data were analyzed using FlowJo software V10 (Tree Star, Ashland, OR, United States).

For western blot analyses, whole UC-MSC (obtained after 6 days of culture in DMEM, Oxium^TM^EXO or commercial medium) and isolated sEV lysates were obtained with RIPA 1× buffer containing 1% w/v of protease inhibitor cocktail (Roche Diagnostics, Mannheim, Germany, Cat. #118735800001). Total protein concentrations were determined with Pierce BCA Protein Assay Kit (Thermo Scientific, Rockford, IL, United States, Cat. #23225), and 2.5 μg of each cell lysate or its corresponding sEV-lysate sample was mixed with Laemmli buffer 5×, heated for 5 min at 95°C, separated on 4–20% gels by SDS-PAGE, and transferred to PVDF membranes (GE Healthcare Limited, Chicago, IL, United States, Cat. #RPN303F). Membranes were blocked for 1 h at RT in Odyssey^®^ Blocking Buffer (LI-COR Biosciences, Lincoln, NE, United States, Cat. #927-400000). Primary antibodies used were Syntenin-1 (1:1000; Novus Biologicals, Centennial, CO, United States, Cat. #NBP2-76873), Flotillin-1 (1:2000; Abcam Inc., Cambridge, MA, United States, Cat. #ab133497), Calnexin (1:2,000; Abcam Inc., Cambridge, MA, United States Cat. #ab22595), and TOMM20 (1:1,000; Novus Biologicals, Centennial, CO, United States, Cat. #NBP2-67501). For fluorescence detection of proteins, Invitrogen^TM^ Goat anti-Rabbit (H + L) Highly Cross-Adsorbed secondary antibody, Alexa Fluor Plus 800, was used (1:25,000; Thermo Fisher Scientific, Waltham, MA, United States, Cat. #A32735). Protein signals were captured using a LI-COR Odyssey^®^ imaging system (LI-COR Biosciences, Lincoln, NE, United States). For probing of other proteins on the same membrane, the membranes were washed three times for 10 min before re-incubation of the next primary antibody.

To verify the sEV structure, isolated sEV samples were visualized using transmission electron microscopy (TEM) as previously described (Zavala et al., 2020). Briefly, solutions of 2 × 10^9^ particles in 12 μL final volume (completed with filtered PBS 1×) for each sample were prepared and deposited on formvar/carbon-coated copper meshes (Electron Microscopy Sciences, Hatfield, PA, United States, Cat. #FCF300-CU) for 1 min, followed by negative staining with 15 μL of 2% w/v uranyl acetate solution for 1 min and dried at RT for 15 min. Imaging was performed at the Advanced Microscopy Facility UMA UC on a Tecnai 12 BioTwin transmission electron microscope (operated at 80 kV; FEI Company, Eindhoven, Netherlands) with iTEM software (Olympus Soft Imaging Solutions GmbH, Münster, Germany). Representative images of each sample were taken at 6,000× and 20,500× magnifications.

Staining of sEV for *in vitro* and *in vivo* tracking was performed with the lipophilic near-infrared fluorescent cyanine dye DiR (Biotium, Fremont, CA, United States, Cat. #60017) as previously described by our group (Rosenberger et al., 2019). Briefly, purified sEV were incubated in the dark for 1 h at 37°C with DiR at a concentration of 71 μM and then washed using MW 3000 size-exclusion exosome spin columns (Invitrogen, Carlsbad, CA, United States, Cat. #4484449) according to the manufacturer’s instructions. After the spin column, the stained particles were analyzed to determine the concentration through NTA as described above. Note that the same volume of incubation was used in the case of PBS + dye controls.

### Secretion Rate of Cell Culture–Derived sEV

Cells were seeded at a density of 6,250 cells/cm^2^ in a six-well plate in a maintenance medium (1 mL/well). Once ∼70% confluence was reached, the culture medium was discarded, and the cells were washed three times with PBS 1× before addition of the induction medium (DMEM or Oxium^TM^EXO or commercial) for sEV secretion. At different time points (2, 4, and 6 days), the supernatant (1 mL) was collected and evaluated directly through NTA to quantify the particles and to determine the mean and mode of particle size, as described above. The particle yield was calculated by dividing the number of particles by the number of seeded cells following the Minimal Information for Studies of Extracellular Vesicles (MISEV) guidelines (Théry et al., 2018).

### Cellular Uptake of sEV

The evaluation of the uptake of isolated sEV generated in the different induction media was carried out as previously described by our group (Alcayaga-Miranda et al., 2016; Rosenberger et al., 2019). In brief, cells were seeded at a density of 10,000 cells/well in a 24-well format. After 24 h, cells were incubated with DiR-stained sEV (3.8 × 10^3^ part/cell) for 6 h at 37°C. As negative controls of the internalization, the experiment was performed at 4°C, and one well at each temperature was incubated with PBS + DiR solution without sEV. To quantitatively measure the exosome uptake, the cells were trypsinized, washed with PBS 1×, and analyzed for DiR signal on the cytometer FACSCanto^TM^ II cytometer (BD Biosciences, San Jose, CA, United States). The data was analyzed using FlowJo software V10 (Tree Star, Ashland, OR, United States).

### *In vivo* Biodistribution Study

C57Bl/6j mice were purchased from Jackson Laboratories (Bar Harbor, ME, United States, Cat. #000664) and maintained at the Cells for Cells animal facility in accordance with protocols revised and approved by the Institutional Animal Care under American Association for Laboratory Animal Science (AALS) training and certification programs. To evaluate *in vivo* the biodistribution pattern of sEV produced in the different culture media, mice (20-week-old male/female) were intraperitoneally (IP) injected with a 100-μL PBS 1× solution containing ∼1 × 10^8^ particles of freshly purified DiR-stained sEV and non-stained sEV (auto-fluorescence control) (*n* = 3 per group). Six hours postinjection, sEV’s fluorescence intensities were assessed using a LI-COR Odyssey imaging system (LI-COR Biosciences, Lincoln, NE, United States) for the entire animal and excised organs, according to the manufacturer’s instructions. As control of the sEV staining procedure, DiR was diluted in 100 μL PBS 1× (at a concentration of 71 μM) and then washed using size-exclusion exosome spin columns.

### Statistical Analysis

Results were expressed as the mean ± SEM values. For *in vitro* data, two-way ANOVA followed by Tukey’s posttest was used for analysis of multiple-comparison groups and two-tailed Student’s unpaired *t*-test to compare two groups. For *in vivo* data, non-parametric tests were used dependently of each case (Kruskal–Wallis or Mann–Whitney). Statistical significance was shown as ^∗^*p* < 0.05; ^∗∗^*p* < 0.01; ^∗∗∗^*p* < 0.001; ^****^p < 0.0001. Error bars represent the standard error of the mean (SEM). The number of data used for the statistical analyses is indicated in the figure legends and corresponds to independent experiments.

## Results

### Proliferation, Cell Surface Markers, and Multipotentiality of UC-MSCs Grown in Oxium^TM^EXO

**FIGURE 1:**
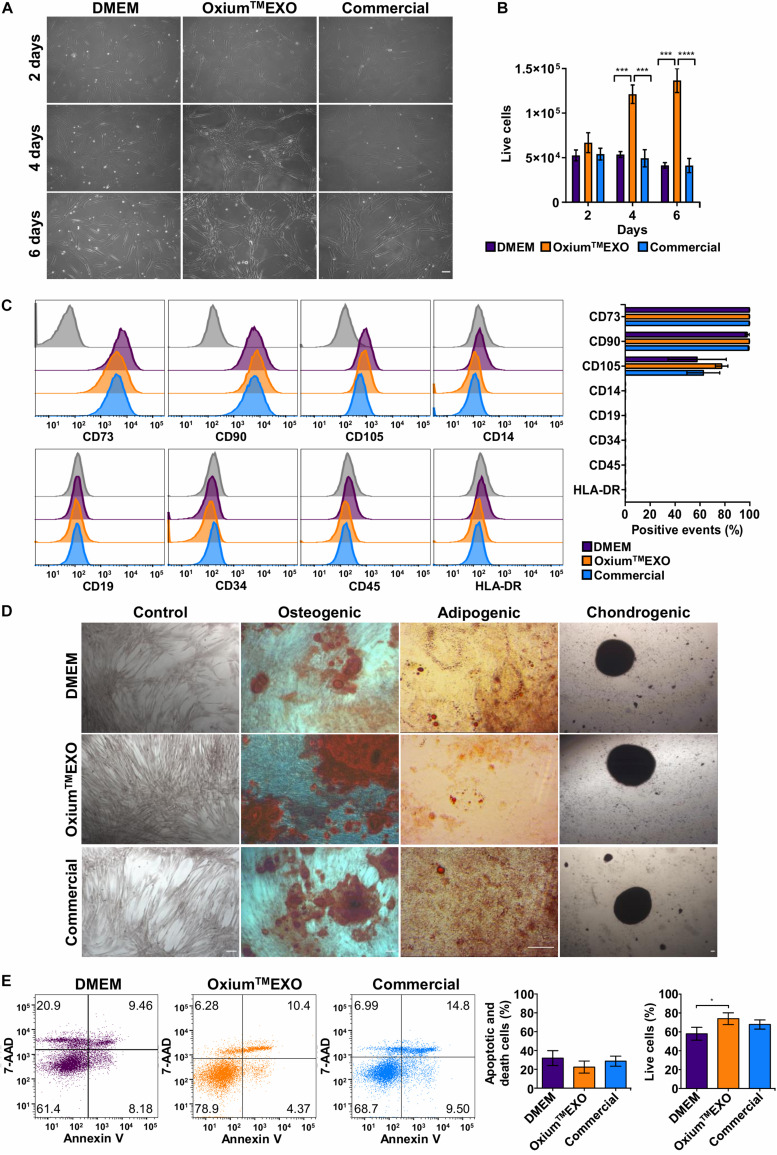
Characterization of UC-MSCs cultured in DMEM, Oxium^TM^EXO, and commercial medium for sEV production. Umbilical cord-derived mesenchymal stromal cells (UC-MSCs) were evaluated at 2, 4, or 6 days post-induction of sEV secretion with DMEM, Oxium^TM^EXO, and commercial medium. **(A)** Microscope images showing cell morphology at the different days post-induction, acquired with an Olympus CKX41 microscope using 10× magnification (scale bar 100 μm). **(B)** After 2, 4, and 6 days post-induction, live cells were counted with the Neubauer chamber. **P < 0.01, ****P < 0.0001, two-way ANOVA, followed by Tukey’s comparison test. **(C)** Flow cytometry analysis of MSCs’ classical surface and purity-control antigens at 6 days post-induction. Histograms of fluorescence intensity for each marker assayed are shown; gray histograms correspond to unstained cell control for each marker. Quantification of positive events for each marker is shown in terms of percentage of total events. **(D)** Multilineage differentiation capacity of UC-MSCs previously cultured for 6 days in the different induction media. Representative images are shown. **(E)** Detection of apoptosis and cell death according to Annexin V/7-AAD staining and its quantification 6 days post-induction. A representative dot plot is shown for each condition. The graphs show mean ± SEM, *n* = 6. *P < 0.05, One tailed t-student.

In order to determine whether xeno-free media for sEV production alter the UC-MSC characteristics, we evaluated their proliferation rate, the expression of cell surface markers, and the tri-differentiation capacity. As displayed in Figure 1A, following 2, 4, or 6 days of culture, we found that at day 4 there is a change in the cell’s morphology only seen in Oxium^TM^EXO, forming a network which continues at least until day 6. Interestingly, from day 4 there is a significant increase in the proliferation of the cells maintained in Oxium^TM^EXO (Figure 1B), reaching at day 6 a mean of 1.36 × 10^5^ live cells, three-fold more in comparison to DMEM (4.13 × 10^4^ live cells) and the commercial medium (4.12 × 10^4^ live cells).

An important aspect of MSCs’ compatible culture condition is the maintenance of their stem features. Through the expression analyses of typical MSC surface antigens such as CD73, CD90, and CD105, the absence of CD14, CD19, CD34, CD45, and HLA-DR (as a MSC culture-purity assessment), plus a multi-lineage differentiation potential assay to osteoblasts, adipocytes, and chondrocytes, we were able to determine the stemness of UC-MSCs after being cultured for 6 days in the different media tested for sEV production. According to flow cytometry analyses, all UC-MSCs showed MSC-proper profiles for the expression of CD73 (>95%) and CD90 (>95%). Meanwhile, CD105 expression was moderately low in all conditions (>50%). As expected, cells grown in the different media showed a very low expression of CD14, CD19, CD34, CD45, and HLA-DR (<2%) (Figure 1C). Next, through a gold standard mesenchymal lineage differentiation protocol, UC-MSCs cultured for sEV production for 6 days in DMEM, Oxium^TM^EXO and a commercial medium retained the ability to differentiate into osteoblasts, adipocytes, and chondroblasts as seen by the morphology and positive staining by Alizarin Red for calcium deposits, by Oil Red O staining for lipid vacuoles and by Safranin O staining for matrix proteoglycans, respectively (Figure 1D). Negative controls for each type of differentiation are shown in Supplementary Figure 2. The present data commensurate the use of Oxium^TM^EXO as a compatible medium for sustaining MSCs’ growth while maintaining their stem cell characteristics *ex vivo*.

### UC-MSCs Cultured in Oxium^TM^EXO Exhibit a Greater Viability Level Following sEV Production Cycle

To verify the viability status of the sEV-producing cells, a flow cytometry analysis of Annexin V and 7-AAD staining was performed (Figure 1E). The cells cultured for 6 days in Oxium^TM^EXO show significantly higher viability (74 ± 13%) and presented less apoptotic and cell death levels (22 ± 14%) in comparison with cells cultured in DMEM (viability = 58 ± 15%; apoptotic and death = 32 ± 17%) or in the commercial medium (viability = 67 ± 10%; apoptotic and death = 28 ± 11%).

### Oxium^TM^EXO Sustains Higher Amounts of Secreted Particles

**FIGURE 2:**
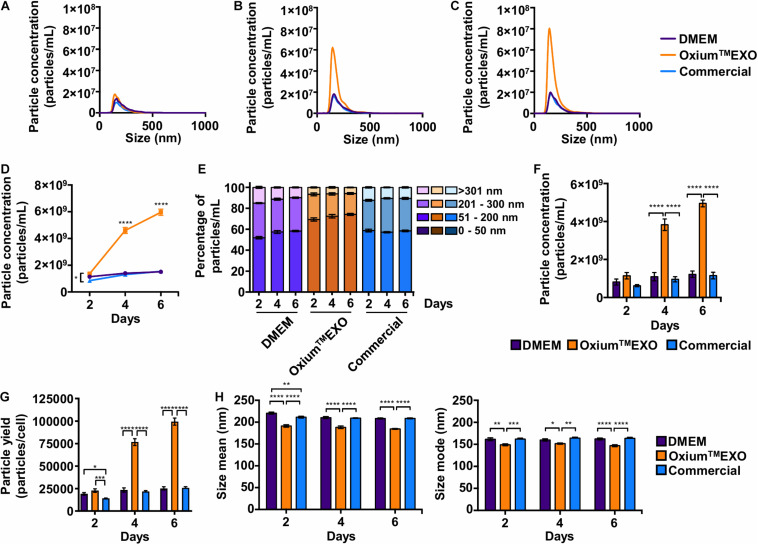
Comparative particle secretion assessment in UC-MSCs cultured in DMEM, Oxium^TM^EXO, and commercial medium for sEV production. Conditioned media were collected at 2, 4, and 6 days post-induction and analyzed by NTA to assess the particle secretion capacity of cells cultured in DMEM, Oxium^TM^EXO, and commercial medium. The graphs show the particles’ concentrations according to their size after **(A)** 2 days, **(B)** 4 days, and **(C)** 6 days post-induction. **(D)** Total particles’ concentration found after 2, 4, and 6 days post-induction with the different media. **(E)** Percentage distribution of particles’ concentrations according to their size: 0–50 nm, 51–200 nm, 201–300 nm, and >301 nm. **(F)** Concentrations of particles in the size range of 51–200 nm. ****P < 0.0001, two-way ANOVA, followed by Tukey’s comparison test. **(G)** Number of particles produced by cells in the different media at 2, 4, and 6 days, respectively. **(H)** Particle size’s mean and mode obtained in the different media at 2, 4, and 6 days, respectively. **P < 0.01, ****P < 0.0001, two-way ANOVA, followed by Tukey’s comparison test. **(A–C)** Graphs show the mean of particle concentrations of five independent-recorded NTA videos. **(D–H)** Graphs show mean ± SEM, *n* = 3.

The key of this study is to set a comparative study, assessing the cell’s sEV secretion rate using different available media. The secreted particles were analyzed directly in the conditioned medium at day 2 (Figure 2A), day 4 (Figure 2B), and day 6 (Figure 2C) post-induction using the Nanoparticle Tracking Analysis (NTA). As displayed in Figures 2A–D, Oxium^TM^EXO induces a greater secretion of particles to the conditioned medium with respect to DMEM and the commercial medium at the different time points evaluated. Specifically, as shown in Figure 2D, at day 2 the cells cultured in Oxium^TM^EXO produced 60% more particles (1.36 × 10^9^ ± 3.49 × 10^8^) than in the commercial medium (8.48 × 10^8^ ± 7.95 × 10^7^), but there was no difference in comparison to DMEM (1.14 × 10^9^ ± 2.47 × 10^8^); at day 4 post-induction, there was a higher particle concentration, over three-fold, in the Oxium^TM^EXO conditioned medium (4.60 × 10^9^ ± 6.80 × 10^8^), which is different from that obtained in DMEM (1.40 × 10^9^ ± 4.31 × 10^8^), and in the commercial medium (1.30 × 10^9^ ± 1.66 × 10^8^); finally, at day 6, the particle concentration continued to increase to almost four-fold in Oxium^TM^EXO (5.96 × 10^9^ ± 7.11 × 10^8^), while no significant particle concentration differences between DMEM (1.51 × 10^9^ ± 3.25 × 10^8^) and commercial conditioned medium (1.54 × 10^9^ ± 2.45 × 10^8^) were observed at this point.

Since the expected size range for sEV varies between 50 and 200 nm, we analyzed further the number of particles that fall under this range (Théry et al., 2018). As seen in Figure 2E, as early as day 2, Oxium^TM^EXO promotes the secretion of particles within the 51–200-nm size range (69 ± 4.6%), enrichment that is maintained through days 4 (72 ± 5.1%) and 6 (74 ± 2.8%) post-induction. In terms of particle concentration, Oxium^TM^EXO induces a higher concentration of particles within the 51–200-nm size range at day 4 (3.82 × 10^9^ ± 9.05 × 10^8^) and day 6 (4.95 × 10^9^ ± 5.33 × 10^8^) in comparison to DMEM (day 4 = 1.09 × 10^9^ ± 6.47 × 10^8^; day 6 = 1.22 × 10^9^ ± 5.01 × 10^8^) and commercial medium (day 4 = 9.61 × 10^8^ ± 4.10 × 10^8^; day 6 = 1.15 × 10^9^ ± 5.19 × 10^8^), as it is shown in Figure 2F. In line with the latter results, the yield of particles per cell was higher in Oxium^TM^EXO (day 2 = 22,765 ± 5,825; day 4 = 76,673 ± 11,344; day 6 = 99,403 ± 11,850) compared to DMEM (day 2 = 19,077 ± 4,124; day 4 = 23,432 ± 7,189; day 6 = 25,203 ± 5,419) and commercial medium (day 2 = 14,137 ± 1,325; day 4 = 21,702 ± 2,781; day 6 = 25,766 ± 4,085), Figure 2G. Interestingly, the overall particles produced in Oxium^TM^EXO showed a smaller size, as seen in both size mean and size mode (Figure 2H). Importantly, these Oxium^TM^EXO advantages are also observed in other types of cells, such as menstrual blood-derived MSCs (Mens-MSCs; Supplementary Figures 3, 5) and fibroblasts (Supplementary Figures 4, 5), as well as in other UC-MSC donors (Supplementary Figure 5), reinforcing the performance of Oxium^TM^EXO.

### Oxium^TM^EXO-Derived Particles Exhibited Standard sEV Characteristics

**FIGURE 3:**
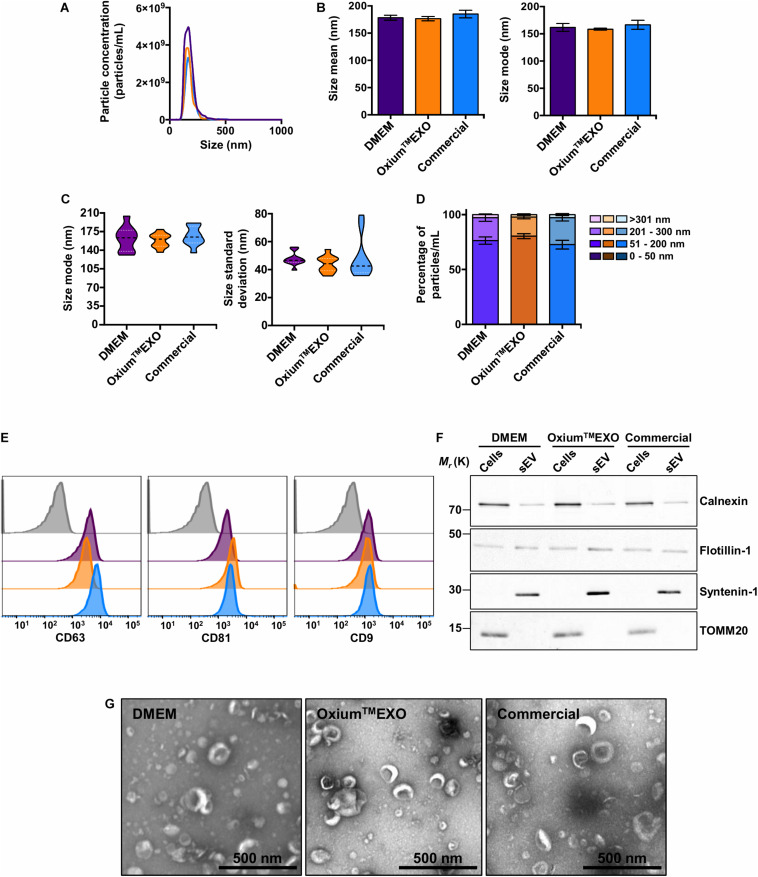
Characterization of isolated UC-MSC-derived sEV produced in DMEM, Oxium^TM^EXO, and commercial medium. The sEV isolated from the different conditioned media were evaluated in terms of particle concentration, size, classical surface/interior markers, and morphology. **(A)** Histogram showing the particles’ concentrations according to their size. Violet line = DMEM-derived sEV; orange line = Oxium^TM^EXO-derived sEV; light blue line = commercial medium-derived sEV. The mean concentration obtained through NTA of five videos for each type of sEV is shown. **(B)** Size’s mean and mode obtained for each type of sEV. **(C)** Size’s mode **(left panel)** and standard deviation data **(right panel)** dispersion. **(D)** Percentage distribution of isolated particles’ concentrations according to their size: 0–50 nm, 51–200 nm, 201–300 nm, and >301 nm. **(E)** Representative histograms of median fluorescence intensity (MFI) obtained by flow cytometry of classical sEV surface markers. Gray = isotype control; violet = DMEM; orange = Oxium^TM^EXO, light blue = commercial medium. **(F)** Western blot, illustrating the presence of the sEV’s membrane-associated protein Flotillin-1 and the sEV’s luminal-scaffold protein Syntenin-1 (involved in sEV’s biogenesis). Note that in the isolated sEV there is minimal or no detectable contamination by Calnexin (endoplasmic reticulum) or TOMM20 (mitochondria), respectively. **(G)** Transmission electron microscopy (TEM) by uranyl acetate negative staining of isolated sEV from ultracentrifuge. The graphs show mean ± SEM. *n* = 2.

With the purpose of characterizing and comparing the quality of sEV produced in the three different culture conditions, we isolated sEV by differential centrifugation, with an additional filtration step of the supernatant prior to ultracentrifugation. This protocol was selected as it represents one of the most widely followed procedures for EV isolation (Théry et al., 2006; Momen-Heravi et al., 2013). The mentioned method allowed the isolation of particles from all three conditioned media, obtaining total particle concentrations in the same order of magnitude of 11 (Supplementary Table 1). The NTA analysis revealed a low concentration of particles obtained from Oxium^TM^EXO (2.96 × 10^11^ ± 1.32 × 10^11^) in comparison to DMEM (4.69 × 10^11^ ± 4.84 × 10^10^), but higher in comparison to the commercial medium (2.73 × 10^11^ ± 5.63 × 10^9^) (Figure 3A), while the size’s mean and mode were similar among the particles obtained in the three media (Figure 3B). It should be noted that the sizes of the particles secreted in Oxium^TM^EXO were more homogeneous than the sizes of those isolated from DMEM or commercial conditioned medium, according to the size’s modes and standard deviation obtained from the NTA video’s analyses (Figure 3C), showing particle size modes ranging from 131.0 to 204.1 nm for DMEM, 136.2 to 179.1 nm for Oxium^TM^EXO, and 135.2 to 191.1 nm for the commercial medium. In contrast to the enrichment of particles in the size range of 51–200 nm observed previously in the analyses performed over those particles present in the non-purified conditioned media, once having applied the isolation method we were not able to distinguish a significant enrichment of particles in the size range of 50–200 nm in the processed Oxium^TM^EXO conditioned medium (Figure 3D). Since the chosen isolation method depends on the operator, we believe that a large-scale, fully automated sEV isolation technique could take full advantage of the Oxium^TM^EXO’s high-quantity and homogeneous-size sEV production, while avoiding particle loss and heterogeneity.

Taking into consideration the International Society for Extracellular Vesicles (ISEV) criteria for EV characterization (Théry et al., 2018), a bead-based flow cytometry analysis of the classical CD63, CD81, and CD9 sEV-surface proteins was performed on the isolated particles. As expected, DMEM-, Oxium^TM^EXO-, and commercial conditioned medium-derived particles presented all of the three sEV typical surface markers CD63 (>90%), CD81 (>90%), and CD9 (>40%) (Figure 3E). Moreover, these particles contained other established sEV markers, such as Flotillin-1 and Syntenin-1 (Figure 3F). Finally, isolated samples contained cup-shaped vesicles of size and morphology consistent with sEV (Figure 3G and Supplementary Figure 6), confirming the EV and exosome nature of the three types of sEV isolated in this work.

### Oxium^TM^EXO-Derived sEV Are Internalized by Target Cells

**FIGURE 4:**
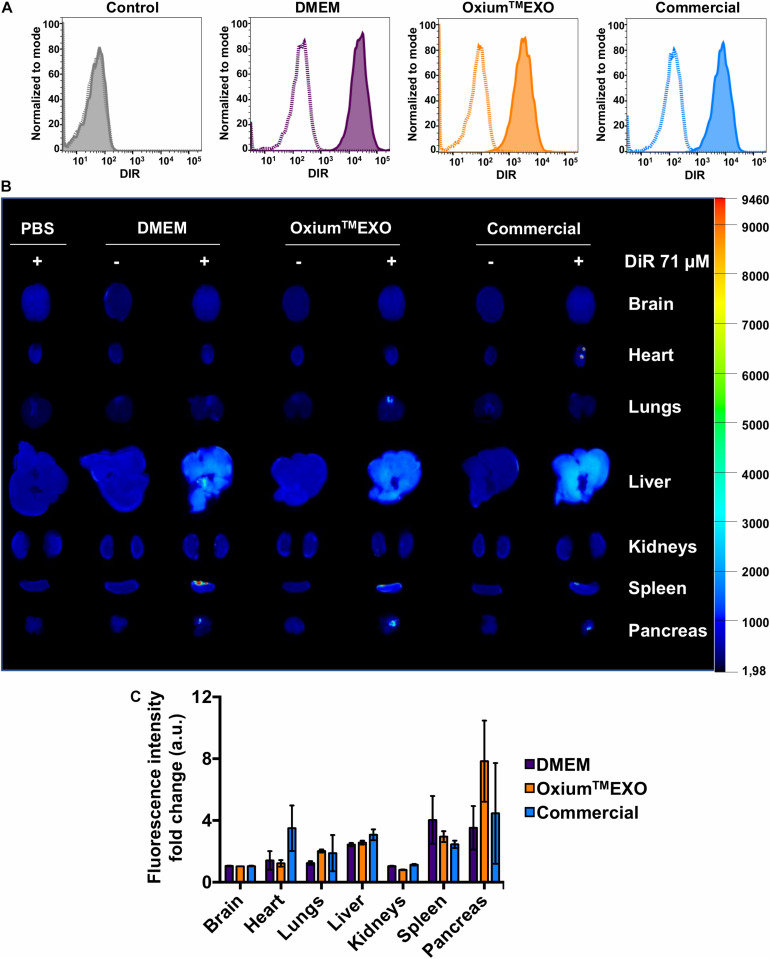
Cellular internalization and *in vivo* biodistribution profile of isolated sEV produced in DMEM, Oxium^TM^EXO, and commercial medium. **(A)** Flow cytometry analysis of MDA-MB-231 cancer cells incubated for 6 h with DiR-stained isolated sEV. Dotted lines = incubation performed at 4°C; solid lines = incubation performed at 37°C; gray histogram = no sEV-incubation control (PBS + DiR); violet histogram = DMEM-derived sEV uptake; orange histogram = Oxium^TM^EXO-derived sEV uptake; light blue histogram = commercial medium-derived sEV uptake. **(B)** Six hours post-injection distribution of DiR-stained isolated-sEV administrated intraperitoneally in mice. **(C)** Fluorescence intensity-fold change observed for each analyzed organ. The fluorescence intensity of DiR-stained sEV-treated mouse organs was normalized with the fluorescence intensity of those organs coming from mice treated with the respective unstained sEV. The graph shows mean ± SEM. *n* = 3 mice for each condition.

To investigate the cellular uptake level of sEV produced under different media, sEV were stained with DiR, a lipophilic dye that fluoresces intensely when inserted into a lipid membrane. Taking into account the promissory use of sEV as therapeutic agents or as drug-delivery vehicles to treat different pathologies but mainly in cancer and osteoarthritis, the MDA-MB-231 cancer cell line and chondrocytes were selected to be incubated with DiR-stained sEV. The stained sEV isolated from DMEM, Oxium^TM^EXO, and commercial conditioned medium were found in all cells, forasmuch as the flow cytometry analyses showed that over 95% of the MDA-MB-231-cultured cells were positive for DiR, implying the cell uptake and internalization of the sEV (Figure 4A, solid lines). As previously described (Rosenberger et al., 2019), a negative control of sEV internalization was done at 4°C, showing no DiR-positive cells neither for DMEM nor for Oxium^TM^EXO or commercial DiR-stained sEV (Figure 4A, dotted lines). The same experimental setup was done using chondrocytes isolated from patients with osteoarthrosis, showing similar results (Supplementary Figure 7). Overall, this result shows that Oxium^TM^EXO-produced sEV maintain their internalization potential by target cells.

### Oxium^TM^EXO-Produced sEV Maintain Their Biodistribution Profile *in vivo*

To assess the sEV biodistribution for determining the main target organs, mice were injected intraperitoneally (IP) with DiR-stained sEV derived from DMEM, Oxium^TM^EXO, or commercial conditioned medium. Once euthanized at 6 h postinjection, the brain, heart, lungs, liver, kidneys, spleen, and pancreas were collected and imaged *ex vivo* (Figures 4B,C). To subtract the possible background noise due to autofluorescence, non-DiR-labeled sEV were administered as a negative control. In addition, to discard the possibility of unspecific staining due to free dye, 100 μl of 1 × PBS was subjected to the DiR-labeling procedure and injected into mice. For both negative controls, no tissue fluorescence was detected, which suggests a reliable signal from tracking DiR-stained sEV and not merely free dye accumulation in the organ’s tissues. Overall, our results show clearly that sEV, independent of their culture condition, display a similar biodistribution pattern. They are able to enter the mouse bloodstream and accumulate after 6 h of IP administration mainly in the liver, spleen, and pancreas, which is in good agreement to previously human sEV biodistribution assays performed in mice (Wiklander et al., 2015).

## Discussion

The potential clinical applications of sEV in regenerative medicine and tissue engineering have gained worldwide interest, which is evident from the number of clinical trials under development. As has been previously reported (Zhao et al., 2020), there are 190 and 56 studies registered in the United States National Institutes of Health clinical trials database^1^ involving exosomes or extracellular vesicles, respectively. Undoubtedly, to meet the clinical expectations of sEV-based therapies, it is essential to resolve the current limitations of low production efficiency and batch inconsistency of clinical-grade sEV. Along these lines, the use of the adequate culture medium for the generation of sEV can stimulate their secretion, hence improving the production process efficiency. Additionally, for clinical-grade production of sEV, it is essential that the culture medium used is xeno- and blood-free of components to comply the regulatory framework, which seeks to avoid the risk of transmission of infectious agents or the elicitation of an immune response in the patient who will receive a sEV-based therapy (United States Food and Drug Administration, 2020).

The results presented here show that the Oxium^TM^EXO medium can support the growth of MSCs to a greater degree than that observed in DMEM or in a commercially available medium that was developed specifically for the collection of sEV. Previous reports showed that xeno-free media can alter the proliferative capacity of human MSCs (Gerby et al., 2017), impacting in their secretory and immunomodulatory properties (Yoshida et al., 2018), or even maintain the MSC characteristics but only when grown imbedded in an extracellular matrix (Rakian et al., 2015). In this work, we assayed for basic cellular functional studies as Oxium^TM^EXO was primarily developed to produce sEV and not as a cell expansion culture medium. Certainly, it has been stated that the metabolic change from FBS-supplemented medium to a xeno-free culture medium impacts the molecular composition including protein, lipid, and miRNA profile changes of purified sEV. This can be explained by the lack of FBS (Li et al., 2015; Haraszti et al., 2019) or by contaminants present in the supplements of the xeno-free medium, altering, for example, the RNA-seq outcomes (Auber et al., 2019). Furthermore, the use of the FBS-depleted medium has also shown altered cellular programs, mainly reducing cell growth and viability (Eitan et al., 2015; Liao et al., 2017; Witwer et al., 2019). Taking into consideration the aforementioned observations, validation studies are required when changing between the cell expansion and sEV production media. The significance and impact of the presumable changes will ultimately depend on the application of interest and in accordance with the expected or desired composition and functionality of the sEV (Witwer et al., 2019). The lower expression observed of CD105 could be attributed to the lack of serum or platelet lysate in the media, which is consistent with previous results (Mark et al., 2013). Notably, the CD105 data dispersion among the replicates was lower in those cells cultured in Oxium^TM^EXO. Also, since sEV are composed of complex macromolecular structures that may induce pleiotropic activities, each specific sEV-based application requires its own functional and potency assays. For instance, some applications are looking for MSC-derived sEV with anti-angiogenic activity, while other sources of MSCs can deliver sEV with pro-angiogenic effects (Alcayaga-Miranda et al., 2016). sEV derived from DMEM, Oxium^TM^EXO, or the commercial medium might not be “one fit for all,” and to avoid data misinterpretation, further tests are required for each of the intended application. The key objective of this comparative study is to determine the advantage of using Oxium^TM^EXO on enhancing the secretion rate of sEV, without modifying its structural, biochemical, and classical properties. This positive impact was determined mainly by the internalization capacity of Oxium^TM^EXO-produced sEV in variable target cells *in vitro* and by the biodistribution profile *in vivo.*

In the current study, the secretion rate of sEV to the supernatant using DMEM, Oxium^TM^EXO, and a commercial medium revealed significant differences in terms of particle accumulation through cell culture days. Oxium^TM^EXO allowed a higher accumulation of particles within the 51- to 200-nm size range, and at least a four-fold increase was measured in comparison to the benchmarked medium. While some authors have suggested that the xeno-free medium positively modulates the secretion rate of sEV (Palamà et al., 2020), achieving a two-fold increase, the comparison was performed by growing the cells in the FBS-free or xeno-free medium, and then sEV production was accomplished by medium changes and maintaining the cells for 72 h in the α-MEM medium until supernatant collection. The supposed FBS-free medium was in fact supplemented with a sEV-depleted FBS, which is still susceptible to be contaminated with FBS-derived sEV, as previously have been demonstrated (Shelke et al., 2014; Lehrich et al., 2018). In the present study, the cells were grown first for 48 h in the hPL-supplemented DMEM and then put through a supplement starvation period of 2, 4, and 6 days before supernatant collection for particle analyses or sEV isolation, achieving with Oxium^TM^EXO the mentioned four-fold increase.

The ultracentrifugation method used here has been reported to have limitations related to upscaling and reproducibility between laboratories, mainly due to the type of rotor (tilting or fixed angle) and its specific parameters (like rotation radius or sedimentation path length) (Livshts et al., 2015). Differences have even been reported at the same laboratory level. Mendt et al. (2018) performed several GMP-compliant sEV isolates for clinical use by ultracentrifugation, obtaining a range of 9.8–15.6 billion exosomes per bioreactor cycle. We believe that the use of other types of sEV isolation techniques, such as ultrafiltration or tangential flow filtration, is recommended to evaluate with greater precision and reproducibility the effect that Oxium^TM^EXO has robustly demonstrated in different cell types prior to isolation. Both ultrafiltration and tangential flow filtration have shown higher sEV isolation efficiency of up to four orders of magnitude over the ultracentrifugation method, maintaining the sEV size and morphology while improving sEV sample purity (Watson et al., 2018; Shu et al., 2020; Tian et al., 2020). These size-based, more automated, and less user-dependent isolation techniques minimize sEV losses, maximize sEV throughput, and allow processing of larger amounts of the conditioned medium.

Also, the sEV may exert their therapeutic or delivery-desired function through the interaction with the acceptor target cells. Herein, isolated and stained sEV from DMEM, Oxium^TM^EXO, and commercially available conditioned media were able to be taken up by human cancer and cartilage cells at 37°C, in accordance with what has been established by the EV community (Russell et al., 2019). Furthermore, these sEV distributed systemically in a healthy mouse model, reaching the liver, spleen, and pancreas, in agreement with previous work in the field (Wiklander et al., 2015; Russell et al., 2019), with no differences at the distribution level among the three types of sEV tested. Taking together, our results imply that the surface markers, cell uptake, and biodistribution pattern of the sEV are not affected by the type of medium used for their production, leaving the quantity of secreted sEV to the supernatant as the essential criterion for favoring the use of one medium over the another, along with the final user’s desired sEV functional potency test.

Finally, clinical translation cannot be achieved without considering investor expectations with regard to the product therapeutic potential, conceivable market authorization, and viability. For this reason, economic factors have been also influencing the field and most importantly the accessibility and affordability of these advanced therapies. Hence, a selection of reagents and procedures that may be applied from preclinical to clinical developments aiming to maintain cell consistency and ultimately reduce manufacturing costs is much needed.

## Data Availability Statement

The original contributions presented in the study are included in the article/Supplementary Material, further inquiries can be directed to the corresponding authors.

## Ethics Statement

The studies involving human participants were reviewed and approved by the Ethics Committee, Universidad de los Andes, Santiago, Chile. The patients/participants provided their written informed consent to participate in this study. The animal study was reviewed and approved by Institutional Animal Care and Use Committee, Universidad de los Andes, Santiago, Chile.

## Author Contributions

FA-M and MK conceived and designed the study. AF-V, CF, YH, and AV-L were responsible for data acquisition and analysis. AF-V, CF, YH, AV-L, FA-M, and MK participated in the manuscript writing and editing. RT-L participated in the Oxium^TM^EXO formulation and manuscript editing. All authors contributed to the article and approved the submitted version.

## Conflict of Interest

MK is the chief scientific officer of Cells for Cells and Consorcio Regenero. MK and RT-L are co-inventors of the patent for the formulation of Oxium^TM^EXO. All other authors received stipends from Consorcio Regenero, a public and private funded Chilean Consortium for Regenerative Medicine.

## Footnotes

1 www.clinicaltrials.gov

2 www.c4c.cl

## Abbreviations

sEV: Small Extracellular Vesicles
DMEM: Dulbecco’s Modified Eagle Medium
UC-MSCs: Umbilical Cord-derived Mesenchymal Stromal Cells
NTA: Nanoparticle Tracking Analysis
OA: Osteoarthrosis.
