# Supplementary material for: A Chemically Defined, Xeno- and Blood-Free Culture Medium Sustains Increased Production of Small Extracellular Vesicles From Mesenchymal Stem Cells
Source: Front Bioeng Biotechnol. 2021 May 26;9:619930. doi: 10.3389/fbioe.2021.619930 (PMC8187876; doi:10.3389/fbioe.2021.619930)
Supplement: Supplementary file 1 [file Data_Sheet_1.PDF]

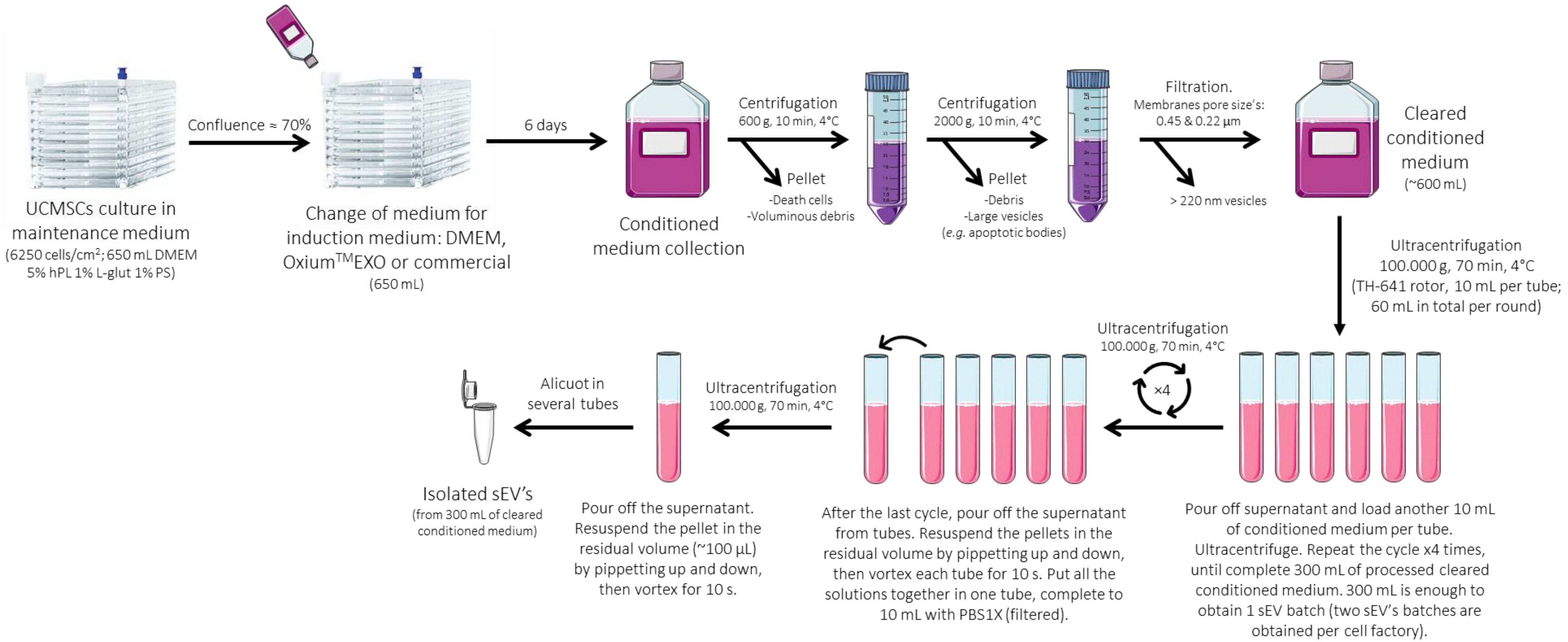

Supplementary Figure 1. sEV production and isolation protocol. Diagram showing the protocol followed for the production and isolation of sEV using the three different mediums evaluated in this work.
