# Supplementary material for: A Chemically Defined, Xeno- and Blood-Free Culture Medium Sustains Increased Production of Small Extracellular Vesicles From Mesenchymal Stem Cells
Source: Front Bioeng Biotechnol. 2021 May 26;9:619930. doi: 10.3389/fbioe.2021.619930 (PMC8187876; doi:10.3389/fbioe.2021.619930)
Supplement: Supplementary file 2 [file Data_Sheet_2.PDF]

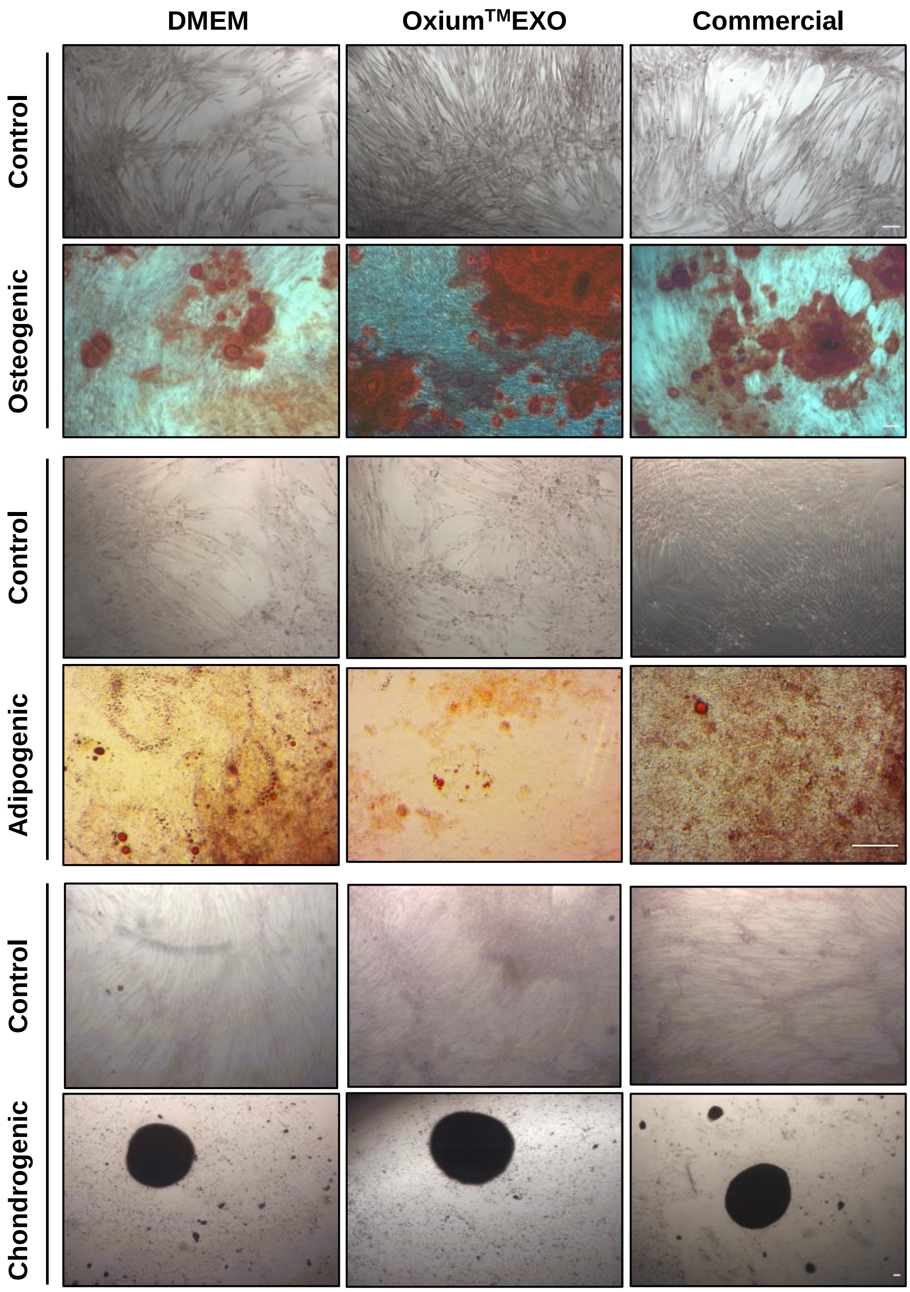

Supplementary Figure 2. Multilineage differentiation capacity assay of UC-MSCs post sEV production cycle. Cells were detached after being cultured for 6 days for sEV production in DMEM (first column), Oxium^{TM}EXO (second column) and commercial medium (third column). Representative images are shown of the osteogenic (upper), adipogenic (middle) and chondrogenic (lower) differentiations, each one with it respective control.
