# Supplementary material for: A Chemically Defined, Xeno- and Blood-Free Culture Medium Sustains Increased Production of Small Extracellular Vesicles From Mesenchymal Stem Cells
Source: Front Bioeng Biotechnol. 2021 May 26;9:619930. doi: 10.3389/fbioe.2021.619930 (PMC8187876; doi:10.3389/fbioe.2021.619930)
Supplement: Supplementary file 3 [file Data_Sheet_3.PDF]

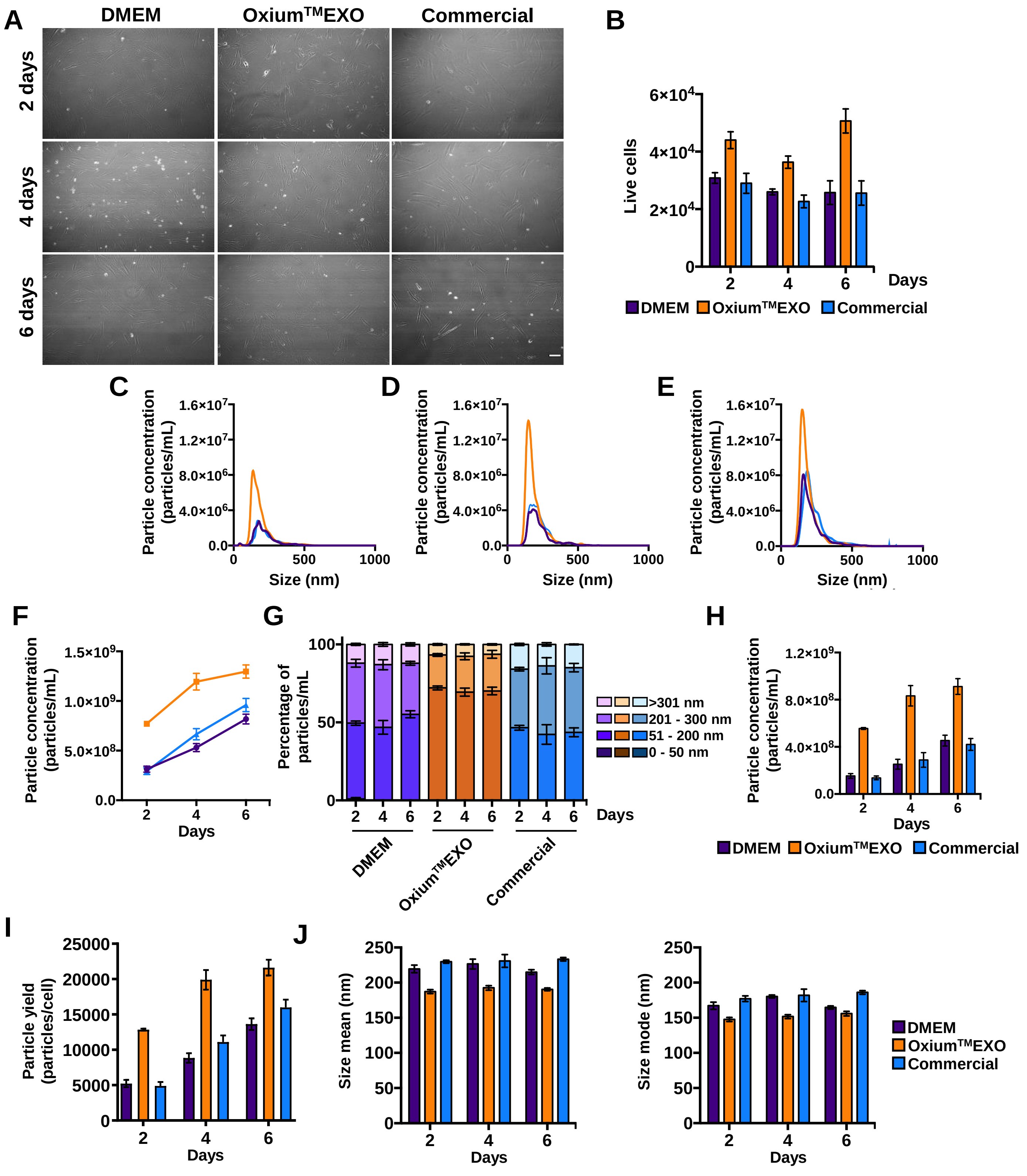

Supplementary Figure 3. Comparative particle secretion assessment in menstrual blood-derived MSCs cultured in DMEM, Oxium^{TM}EXO and commercial medium for sEV production. Conditioned medium was collected 2-, 4- and 6 days post-induction and analyzed by NTA to assess the particle secretion capacity of cells cultured in DMEM, Oxium^{TM}EXO and commercial medium. (A) Microscope images showing cell morphology at the different days post-induction, acquired with an Olympus CKX41 microscope using 10x magnification (scale bar 100 µm). (B) After 2, 4 and 6-days post-induction, live cells were counted with Neubauer chamber. The graphs show particle's concentration according to their size after (C) 2 days, (D) 4 days and (E) 6 days post-induction. (F) Total particle's concentration found after 2-, 4- and 6-days post-induction with the different mediums. (G) Percentage distribution of particle's concentration according to their size: 0-50 nm; 51-200 nm; 201-300 nm and >301 nm. (H) concentration of particles in the range size of 51-200 nm. (I) Particle yield obtained in the different mediums at 2, 4 and 6 days, respectively. (J) Particle size's mean and mode obtained in the different mediums at 2, 4 and 6 days, respectively. (C), (D) and (E) graphs show the mean of particle concentration of 5 independent-recorded NTA videos. (B), (F), (G), (H) and (I) graphs show mean ± SEM, n=1 biological replicate (with 3 technical replicates).
