# Supplementary material for: A Chemically Defined, Xeno- and Blood-Free Culture Medium Sustains Increased Production of Small Extracellular Vesicles From Mesenchymal Stem Cells
Source: Front Bioeng Biotechnol. 2021 May 26;9:619930. doi: 10.3389/fbioe.2021.619930 (PMC8187876; doi:10.3389/fbioe.2021.619930)
Supplement: Supplementary file 5 [file Data_Sheet_5.PDF]

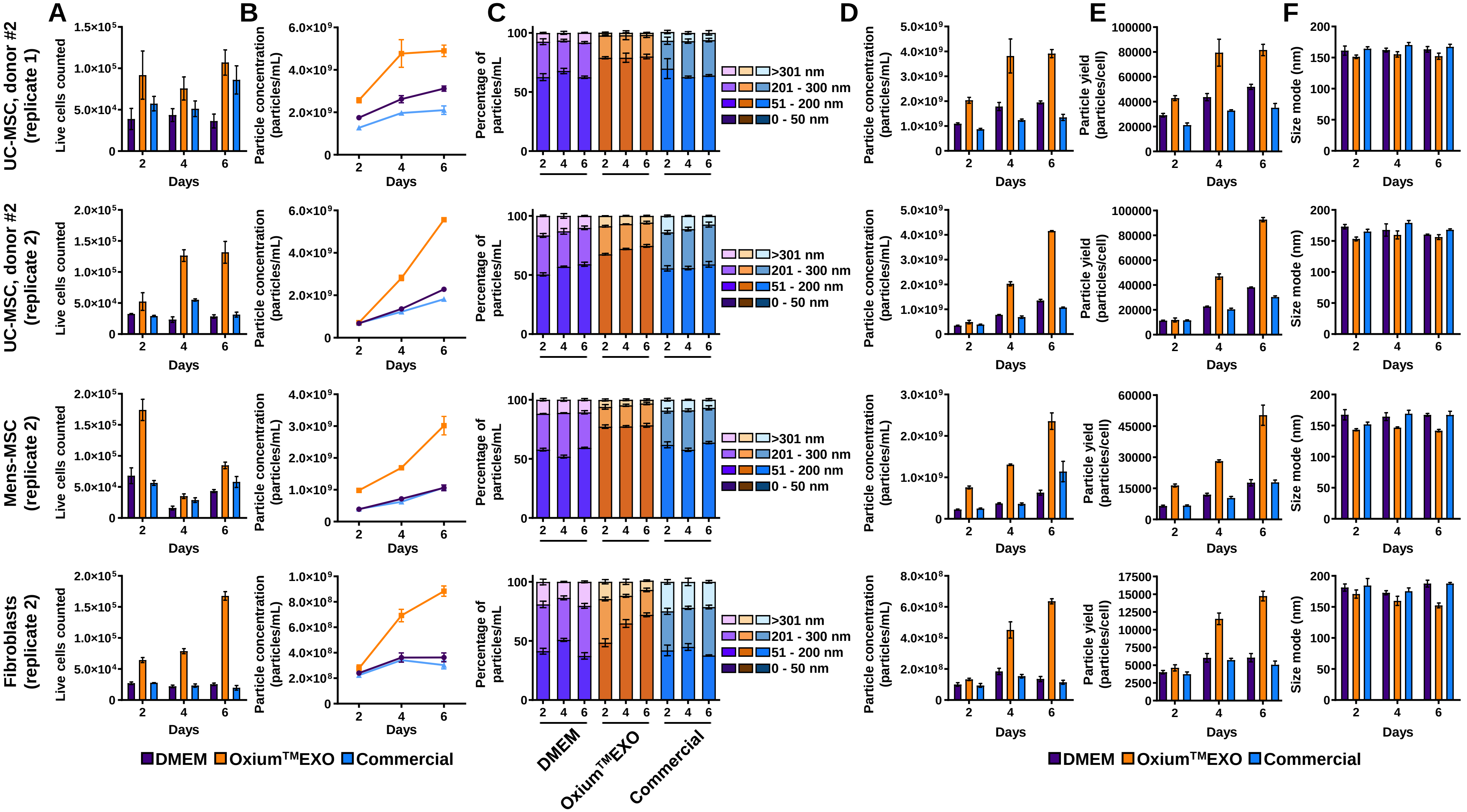

Supplementary Figure 5. Comparative particle secretion assessment in umbilical cord-derived MSCs, menstrual blood-derived MSCs and fibroblasts cultured in DMEM, Oxium^{TM}EXO and commercial medium for sEV production. Conditioned medium was collected 2-, 4- and 6-days post-induction and analyzed by NTA to assess the particle secretion capacity of UC-MSCs from a different donor (first and second rows; corresponding to two independent biological replicates), Mens-MSCs (third row; corresponding to a second independent biological replicate) and fibroblasts (fourth row, corresponding to a second independent biological replicate), cultured in DMEM, Oxium^{TM}EXO and commercial medium. (A) After 2, 4 and 6-days post-induction, live cells were counted with Neubauer chamber. (B) Total particle's concentration found after 2-, 4- and 6-days post-induction with the different mediums. (C) Percentage distribution of particle's concentration according to their size: 0-50 nm; 51-200 nm; 201-300 nm and >301 nm. (D) concentration of particles in the range size of 51 to 200 nm. (E) Number of particles produced by cells in the different media at 2, 4 and 6 days, respectively. (F) Particles size's mode obtained in the different mediums at 2, 4 and 6 days, respectively. (A), (B), (C), (D) (E) and (F) graphs show mean ± SEM, n=1 biological replicate (with 3 technical replicates each).
