# Supplementary material for: A Chemically Defined, Xeno- and Blood-Free Culture Medium Sustains Increased Production of Small Extracellular Vesicles From Mesenchymal Stem Cells
Source: Front Bioeng Biotechnol. 2021 May 26;9:619930. doi: 10.3389/fbioe.2021.619930 (PMC8187876; doi:10.3389/fbioe.2021.619930)
Supplement: Supplementary file 6 [file Data_Sheet_6.PDF]

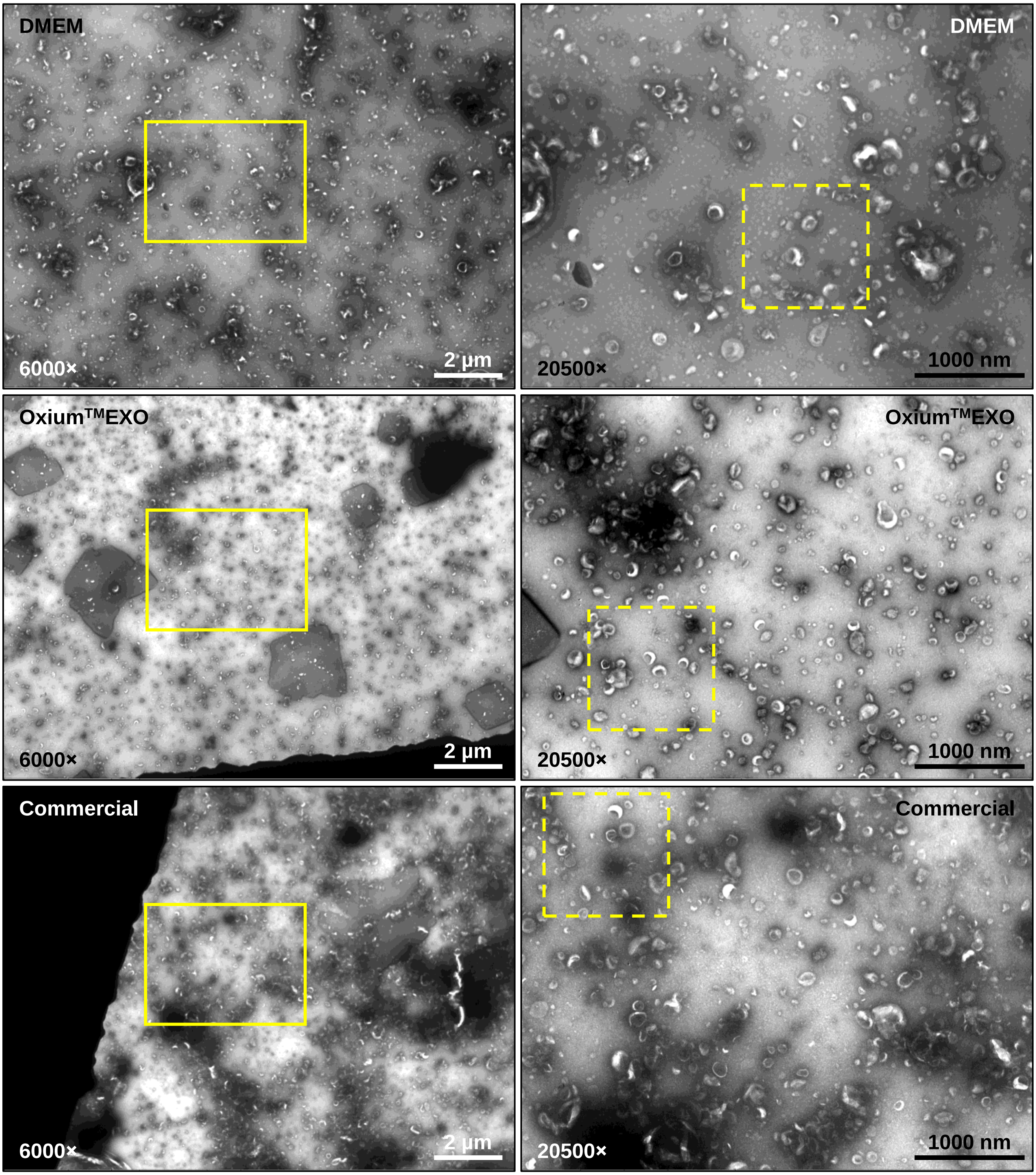

Supplementary Figure 6. Transmission electron microscopy analysis of isolated sEV samples. Representative wide-field images taken at a magnification of 6,000× (left) or 20,500× (right) of DMEM- (upper), Oxium^{TM}EXO- (middle) or commercial- (lower) isolated sEV samples, respectively. Solid yellow lines indicate the area selected for visualization at 20,500×; dashed yellow lines indicate the image area shown in Figure 3G.
