# Supplementary material for: A Chemically Defined, Xeno- and Blood-Free Culture Medium Sustains Increased Production of Small Extracellular Vesicles From Mesenchymal Stem Cells
Source: Front Bioeng Biotechnol. 2021 May 26;9:619930. doi: 10.3389/fbioe.2021.619930 (PMC8187876; doi:10.3389/fbioe.2021.619930)
Supplement: Supplementary file 7 [file Data_Sheet_7.PDF]

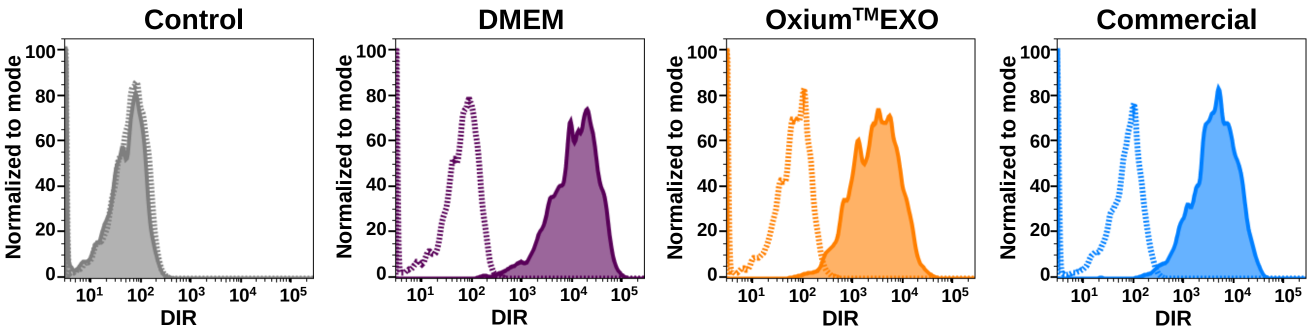

Supplementary Figure 7. Human OA chondrocytes internalization of isolated sEV produced in DMEM, Oxium^{TM}EXO and commercial medium. Flow cytometry analysis of human OA chondrocytes incubated for 6 h with DiR-stained isolated-sEV. Dotted lines = incubation performed at 4°C; solid lines = incubation performed at 37°C; grey histogram = no sEV-incubation control; violet histogram = DMEM-derived sEV uptake; orange histogram = Oxium^{TM}EXO-derived sEV uptake; light blue = commercial medium-derived sEV uptake.
