# Supplementary material for: A Chemically Defined, Xeno- and Blood-Free Culture Medium Sustains Increased Production of Small Extracellular Vesicles From Mesenchymal Stem Cells
Source: Front Bioeng Biotechnol. 2021 May 26;9:619930. doi: 10.3389/fbioe.2021.619930 (PMC8187876; doi:10.3389/fbioe.2021.619930)
Supplement: Supplementary file 8 [file Data_Sheet_8.PDF]

	Isolation replicate	Particle concentration (particles/mL)	Protein content (µg/µl)	Particles/µg of protein
**DMEM**	1	5.03×10^{11}	---	---
	2	4.35×10^{11}	0.81	5.38×10^{8}
**Oxium^{TM}EXO**	1	2.03×10^{11}	---	---
	2	3.89×10^{11}	0.49	7.96×10^{8}
**Commercial medium**	1	2.77×10^{11}	---	---
	2	2.69×10^{11}	0.60	4.50×10^{8}

Supplementary Table I. Total particle concentration obtained after sEV isolation and protein quantification. Two independent isolations were performed from DMEM, Oxium^{TM}EXO and commercial conditioned medium collected 6 days post-induction. Both replicates were obtained from the same collected supernatant. The quantified samples were used in western blots and TEM analyses.
